# First person – Sriikhar Vedurupaka and Bita Jadali

**DOI:** 10.1242/bio.062502

**Published:** 2026-02-18

## Abstract

First Person is a series of interviews with the first authors of a selection of papers published in Biology Open, helping researchers promote themselves alongside their papers. Sriikhar Vedurupaka and Bita Jadali are co-first authors on ‘ A protocadherin mediates oral placode morphogenesis in the tunicate *Ciona*’, published in BiO. Sriikhar is an undergraduate researcher in the lab of Dr Alberto Stolfi at Georgia Institute of Technology, Atlanta, GA, USA, investigating the neurodevelopment of model organisms and their cellular and molecular mechanisms. Bita is a PhD student in the same lab, investigating the mechanisms and signalling of neural death during tunicate metamorphosis.


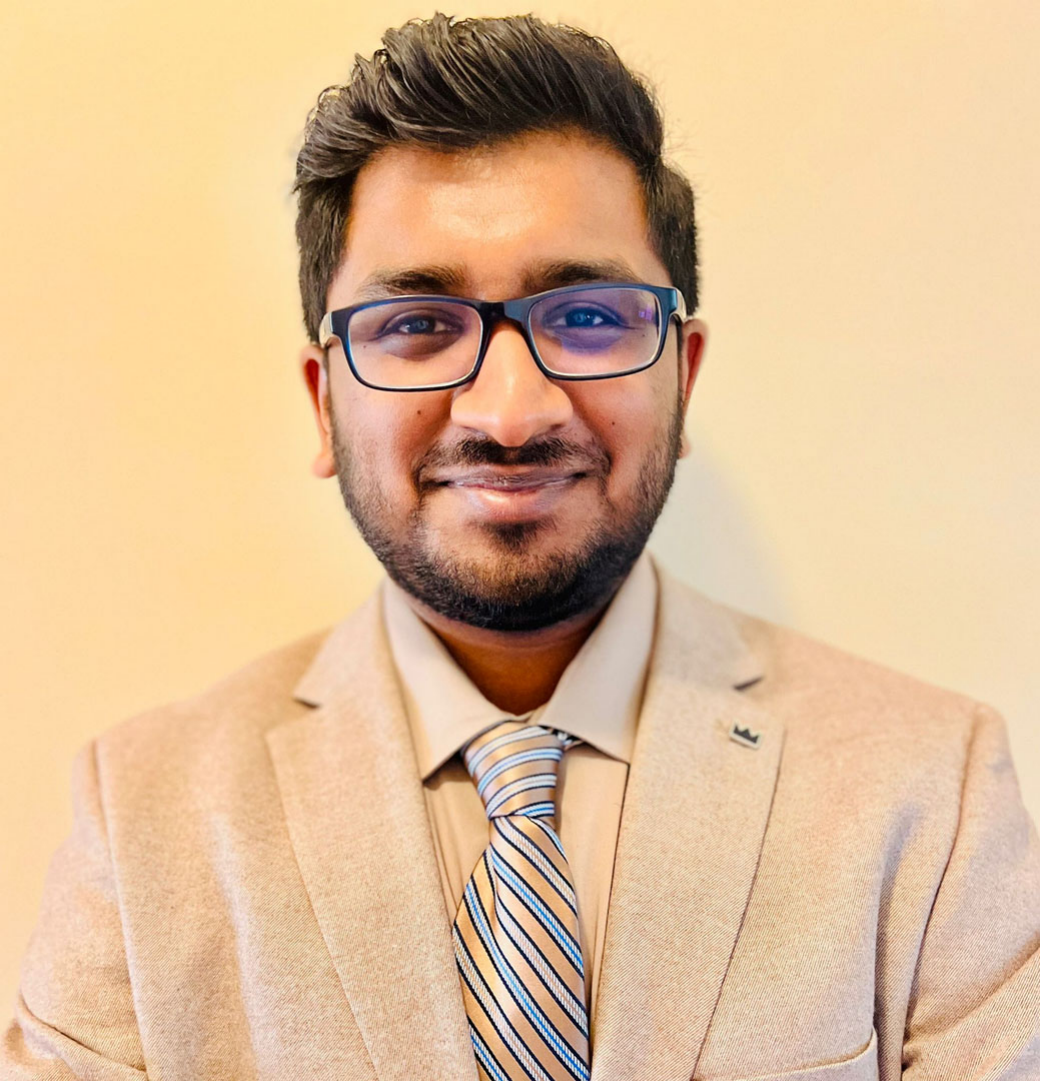


**Sriikhar Vedurupaka**

**Describe your scientific journey and your current research focus**

**S.V.:** I graduated from the Forsyth Central High School STEM Biotechnology Program in 2020, where I developed strong laboratory skills and a disciplined approach to scientific problem solving that prepared me well for collegiate research. Through this program, I gained hands-on experience with techniques ranging from agarose gel electrophoresis and bacterial staining to *Caenorhabditis elegans* model organism research, RNA interference and CRISPR-based approaches. These early exposures, combined with a growing interest in the neurosciences, motivated me to pursue research that bridges molecular tools with questions about nervous system function. This trajectory led me to join Dr Stolfi's lab as an undergraduate researcher, where I could apply and deepen my technical training in a rigorous research environment. In particular, I was drawn to tunicates as a model system, believing that their unique biology and nervous system organization offer valuable insights for advancing our understanding of neuroscience.

**B.J.:** While I ultimately graduated from the University of Georgia majoring in biology, I actually initially started out as undecided with no idea where my degree would take me. I had always enjoyed learning about how the body, especially the brain, works, so I had toyed with the idea of pursuing medicine among other things, but after some encouragement from my friends and support from my past professors, I applied to grad school during my gap year working at the library and started my PhD in Biology at the Georgia Institute of Technology soon after that. Even though I had no idea what a tunicate really was when starting out, I instantly felt at home during my rotation in Dr Alberto Stolfi's lab, where we study neurodevelopment in the tunicate *Ciona robusta*, a model organism I've grown really fond of. Tunicates are invertebrate chordates that undergo metamorphosis from a motile larva to immobile adult; so, for my thesis research, I'm specifically focused on the mechanisms that signal death of their larval nervous system during this metamorphosis as well as the signals that protect their adult neural progenitors from this degeneration.

**Who or what inspired you to become a scientist?**

**S.V.:** From an early age, I was driven by curiosity about how the world works, which led me to participate in science workshops, robotics clubs and health fairs throughout elementary and middle school. My time in the Forsyth Central High School STEM Academy helped me realize that science provides a language for understanding the mechanisms underlying life, transforming curiosity into structured inquiry. As I developed this foundation, I became motivated to pursue a deeper and more rigorous understanding of biological systems. In particular, my fascination with the brain, consciousness and the fundamental questions of the human condition drew me toward neuroscience. This interest ultimately led me to join the Stolfi lab, an experience for which I remain deeply grateful for shaping my identity as a scientist.

**B.J.:** Being a voracious reader growing up, I've always loved to learn about most everything and anything, and my parents nurtured this love of learning by providing an environment full of library and museum visits, PBS documentaries on TV and much more. In college, my passion for biology as a whole slowly grew through my classes – such as cell biology, physiology and gerontology – and, as a part of my degree, I was required to take a research-project lab course, which really was my first small taste of research. I actually never really envisioned myself becoming a scientist as I applied to graduate school mostly with higher education teaching in mind and never thought myself cut out for being a scientist, but my time here at Tech has really made me fall in love with scientific research, and I've learned so much.

**How would you explain the main findings of your paper?**

Here, we were specifically interested in a small circle, or ‘rosette’, of cells in the *Ciona* larva that survives metamorphosis and gives rise to the mouth of the adult. Adhesion molecules are proteins that help cells stick to one another, and one such adhesion molecule [Protocadherin.e (Pcdh.e)] was found to be specifically present in these future mouth cells. We hypothesized that Pcdh.e might be helping this rosette of cells stick tightly to later form a properly shaped adult mouth in the right place. Our results showed that Pcdh.e was indeed important in keeping these cells together, as the rosette was disrupted when Pcdh.e was removed. This also affected proper formation of the adult mouth, as predicted. Additionally, Pcdh.e seems to function similarly to how our own protocadherin proteins work, and genes involved in the development of our own mouth are also important for controlling the production of Pcdh.e in these future mouth cells in *Ciona*. So, by learning more about Pcdh.e in *Ciona*, we might be able to better understand how our own mouth forms!

**What are the potential implications of this finding for your field of research?**

There is still so much unknown about how the future mouth forms in *Ciona*, but this paper set out to figure out one piece of this puzzle, which is how Pcdh.e might be involved in keeping these cells together. Overall, our findings help open the door to understanding, in both *Ciona* and vertebrates, how different genes control the formation of the mouth, and the potential role of adhesion molecules in keeping this and other groups of cells together during development.


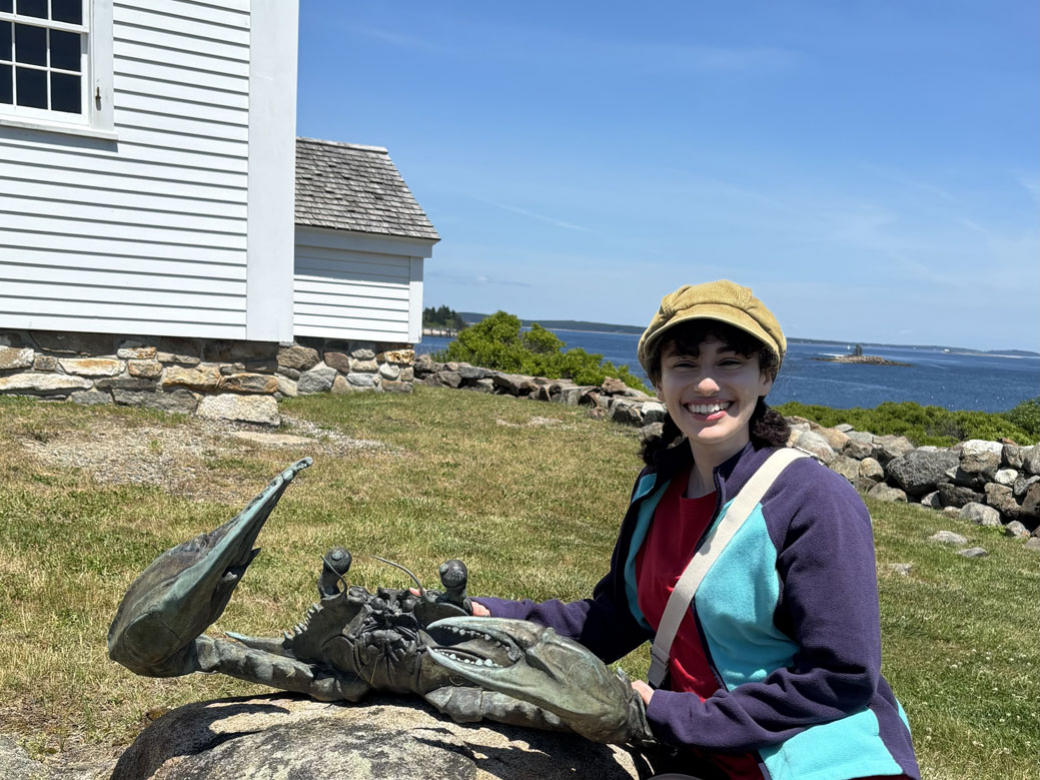


**Bita Jadali**

**Figure:**
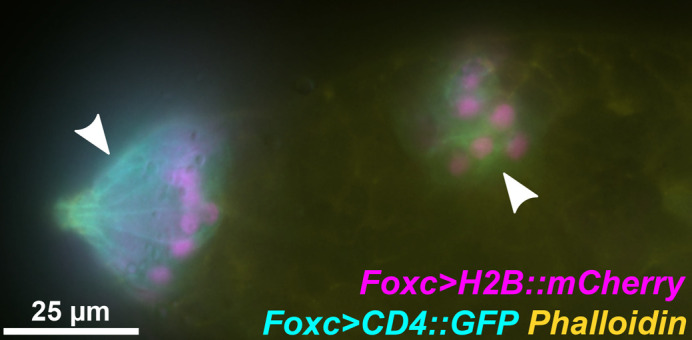
**The head of a tunicate *Ciona robusta* larva with fluorescent reporters marking the papillae (left arrowhead) and rosette of cells that give rise to the adult mouth (right arrowhead).** Both structures arise from the same original population of cells during early development before branching off into their two distinct groups. Cyan, cell membrane reporter; magenta, cell nucleus reporter.

**Which part of this research project was the most rewarding?**

**S.V.:** The most rewarding aspect of the Pcdh.e project was seeing it through to completion, culminating in an accepted manuscript in Biology Open, an achievement made possible through close collaboration with my co-first author, Bita, whose contributions were instrumental in advancing the work. As an undergraduate, experiencing the full scientific process from hypothesis formation and experimental design to data analysis and interpretation was deeply satisfying and formative. I am especially grateful for the training and mentorship I received from Sydney Popsuj, PhD, Christopher Johnson, PhD and our PI, Dr Alberto Stolfi, whose guidance and trust allowed me to take true ownership of the project. That trust transformed the work from a task into a responsibility, giving me the confidence to think and act independently as a scientist. Ultimately, the opportunity to function as a true neuroscientist, rather than simply a trainee, is what I will remember most.

**B.J.:** As I started out in the Stolfi lab, this project was a sort of ‘training project’ for me as I began to get acquainted with our experimental techniques and model system. So, while working on this project, I experienced a lot of ‘learning opportunities’ along the way. But these failures made it even more worth it when I finally succeeded and got data! It was also very satisfying to see the project grow into telling a more comprehensive story through the further contributions I made during the revision process.

… by learning more about Pcdh.e in *Ciona*, we might be able to better understand how our own mouth forms!

**What do you enjoy most about being an early-career researcher?**

**S.V.:** What I enjoy most about being an early-career researcher is the chance to learn deeply while still having the freedom to ask fundamental questions and explore ideas without fear of being wrong. My time in Dr Stolfi's lab has brought me close to the experimental process, where I can see how hypotheses evolve into results and interpretation. The experience allowed me to grow through mentorship while gradually developing independence and ownership over my work. Overall, it is rewarding to build a strong foundation in rigor, curiosity and scientific judgment early on.

**B.J.:** Even though it's only been a few years since I started as a PhD student, I'm surprised how much I've come along as a scientist and researcher. I've learned so much since first starting out. But I also love how there's still so much out there for me to learn and experience as an early-career researcher. It's so exciting that there's still so many more ‘firsts’ out there for me to achieve (though I got my ‘first’ paper achievement with this publication), and I can't wait to experience and learn more!

**What piece of advice would you give to the next generation of researchers?**

**S.V.:** I would encourage the next generation of researchers to stay curious and enjoy the process, because this is an incredibly exciting time to be a scientist. Rapid advances in biotechnology, engineering, artificial intelligence and related fields have created unprecedented opportunities for discovery, and curious minds are essential to pushing new boundaries. Equally important is persistence – meaningful research often takes years, as our Pcdh.e project did, but contributing even a small piece to the growth of a field makes the effort worthwhile. Ultimately, progress in science comes from sustained curiosity paired with the willingness to keep going when challenges arise.

**B.J.:** While science is usually thought to be independent and isolating work, the people you surround yourself with are so important. I wouldn't have grown into the researcher I currently am without my wonderful labmates and PI, who have created such a welcoming environment for me to thrive in as a scientist. For example, Sydney Popsuj, a former grad student from our lab, who’s currently a postdoc at the Davidson lab at Swarthmore College, really mentored and helped me not only learn ‘how to be a PhD student’ but research wise as well, from originally pitching the idea to go after Pcdh.e to then helping connect the two research threads after Sriikhar graduated and I began and then supervising both of us on the project. It really takes a village to become a good researcher, so finding yourself a good support and mentorship network where you can ask for help and guidance is very important, and, in the long run, collaboration also helps the scientific research side of things as well.

… progress in science comes from sustained curiosity paired with the willingness to keep going when challenges arise

**What's next for you?**

**S.V.:** I graduated from the Georgia Institute of Technology in the Spring of 2024 with a Bachelor's of Science degree in Neuroscience and double minors in Health and Medical Sciences and Philosophy. I am currently working in the medical field and am hoping to join medical school and become a physician-scientist.

**B.J.:** In the grand scheme of things, after finishing my PhD, I ultimately hope to stay in academia and maybe one day become a professor as I enjoy teaching as much as I love research. But in the immediate future, as I'm only halfway through my PhD, I hope to develop my own thesis project further in the meanwhile.
